# Functional stroke outcomes after mobile stroke unit deployment – the revised protocol for the Berlin Prehospital Or Usual Delivery of acute stroke care (B_PROUD) part 2 study

**DOI:** 10.1186/s42466-019-0022-4

**Published:** 2019-06-03

**Authors:** Peter Harmel, Martin Ebinger, Erik Freitag, Ulrike Grittner, Irina Lorenz-Meyer, Ira Napierkowski, Christian H. Nolte, Bob Siegerink, Heinrich J. Audebert

**Affiliations:** 1 Department of Neurology, Charité - Universitätsmedizin Berlin, Hindenburgdamm 30, 12203 Berlin, Germany; 2 Center for Stroke Research, Charité - Universitätsmedizin Berlin, Berlin, Germany; 3 Department of Neurology, Medical Park Berlin Humboldtmühle, Berlin, Germany; 4 Institute of Biometry and Clinical Epidemiology, Charité - Universitätsmedizin Berlin, Berlin, Germany; 5 Berlin Institute of Health (BIH), Berlin, Germany; 6 German Centre for Cardiovascular Research (DZHK), Partner Site, Berlin, Germany; 7 German Center for Neurodegenerative Diseases (DZNE), Partner Site, Berlin, Germany

**Keywords:** Stroke, Prehospital, Mobile stroke unit, Thrombolysis, Endovascular thrombectomy, Functional outcome

## Abstract

**Background:**

Studies investigating the Mobile Stroke Unit (MSU) concept have shown increased thrombolysis rates, reduced alarm-to-treatment times and improved prehospital triage. Yet, so far, there is no definite scientific proof of better functional outcome after MSU deployment compared to regular ambulances.

**Methods:**

We provide a revised protocol for the second part of the B_PROUD trial as organization of the MSU dispatch did not meet the anticipated standards in the first part. B_PROUD is a pragmatic, prospective study comparing functional outcomes of treatment candidates with or without MSU care. Treatment candidates are defined as patients with a final diagnosis of ischemic stroke or transient ischemic attack, onset-to-dispatch-times ≤4 h, disabling symptoms not resolved at time of ambulance arrival, and the ability to ambulate prior to the qualifying event. These patients are included if their emergency call prompted a stroke alarm at the dispatch center during MSU operation hours (7 am–11 pm, Monday-Sunday) and if the emergency is located within the MSU operation area in Berlin, Germany. The intervention group consists of patients who are cared for by the MSU. When the MSU is already in operation for another emergency, MSU dispatches are handled by regular ambulances (about 45%). These dispatches create the control group. Blinded stroke physicians assess the modified Rankin Scale (mRS) score in recorded structured interviews 3 months after stroke. The primary outcome is the degree of disability and death over the full range of the mRS. As a change to the previously published protocol and only pertinent in case of more than 9% lost-to-follow-up, a co-primary outcome was introduced consisting of the proportions of death, new institutional care or severe disability in patients with additional use of information from registration offices.

**Perspective:**

The results will inform parties involved in acute stroke care organization on the effectiveness of the MSU concept.

**Trial registration:**

The protocol is registered in (NCT03931616) and has been approved by the ethical review committee of the Charité – University Medicine Berlin (EA4/109/15) on September 2, 2015. The study protocol of B_PROUD part 1 had been published in the International Journal of Stroke as “Berlin Prehospital Or Usual Delivery of acute stroke care (B_PROUD) – study protocol” (doi: 10.1177/1747493017700152) on March 22, 2017 [1] previous to first patient’s registration.

## Background

In the event of an acute ischemic stroke, fast recanalization of the occluded vessel is crucial because of the rapid death of neurons in ischemic brain tissue [2]. The only evidence-based measures to achieve tissue reperfusion are administration of tissue-type plasminogen activator and endovascular thrombectomy. The Mobile Stroke Unit (MSU) concept was first introduced in 2008 in Homburg, Germany [3]. In 2011 the Stroke Emergency Mobile (STEMO) was implemented in Berlin, Germany and yielded clear improvements regarding thrombolysis rates and onset-to-treatment times [4]. Additionally, ischemic and hemorrhagic stroke patients have been more accurately routed to hospitals with Stroke Units or neurosurgery departments [5]. In the meantime, the MSU concept has been implemented in many cities worldwide, and various groups contribute their experiences to the Pre-hospital Stroke Treatment Organization (PRESTO) [6]. Yet, despite a non-significant trend for disability-free survival at 3 months in favor of the MSU care group in a registry-based analysis [7, 8], definite scientific proof of a positive effect of MSU treatment on functional outcome of stroke patients is still lacking. Thus, we started the B_PROUD study on February 1, 2017 [1]. In the meantime, the STEMO service in Berlin was extended to three simultaneously operating STEMOs covering almost the entire Berlin city area. Since logistical problems occurred during almost the entire first study period, we recognized the necessity of a revised study protocol for an additional evaluation during the period after resolving these challenges. The deviations from the original STEMO setting during the PHANTOM-S trial [4] included reduced accuracy of dispatching for stroke emergencies, fewer STEMO dispatches for severe stroke cases (because general emergency physicians were dispatched to patients with impaired consciousness or instable vital parameters instead of the STEMO team), more frequent cancellations of the STEMO dispatch (thus leading to more cross-overs and in-hospital thrombolyses), and longer distances to scene. These issues led to fewer and more delayed STEMO interventions and thrombolytic treatments. After adjustment of the STEMO dispatch organization, we expect that the use of the dispatch algorithm will be equivalent to its use during the preceding PHANTOM-S trial. Following the recommendation of the B_PROUD Data Safety Monitoring Board and in agreement with the Berlin Department of Internal Affairs, we decided to start a second part of the B_PROUD study - with full reporting of the first evaluation period.

## Methods

### Aim of the trial

Here, we describe a confirmatory trial to prove the efficacy of the MSU intervention compared to regular care using the modified Rankin Scale (mRS) score 3 months after event.

### Study description and study design

B_PROUD (Berlin Prehospital Or Usual Delivery of acute stroke care) is a pragmatic, prospective, multicenter study with blinded outcome assessment (PROBE design). Inclusion of eligible patients is currently carried out in the metropolitan area of Berlin, Germany, in cooperation with all 15 stroke centers of various Berlin hospital owners. The B_PROUD study makes use of the B-SPATIAL registry (Berlin – specific acute therapy in ischemic or hemorrhagic stroke with long-term follow-up, NCT03027453) that collects 3 months follow-up assessment on an opt-out basis. In line with German data-protection legislation and approval of the Berlin data protection representatives, patients are informed beforehand about the planned follow-up and can opt-out at any time before or during the telephone interview or optional questionnaire based assessment.

### Arms and intervention

The intervention group consists of patients for whom an MSU is deployed by the dispatch center of the Berlin Fire Department after stroke suspicion during emergency call [9]. Based on the preceding PHANTOM-S trial [4], approximately 45% of the stroke alarms are expected to be handled by regular ambulances because the MSU is already in operation or undergoing service. Compared to regular ambulances offering standard of care, the intervention by MSU includes prehospital neurovascular expertise by a neurologist staffing the MSU, computed tomography (CT) based brain scanning including visualization of large vessel occlusion by CT-angiography, and specific pre-notification to endovascular treatment capable centers. Details of the STEMO equipment, staffing and operational procedures have been published elsewhere [10, 11]. After a decision of the Berlin state government that STEMO care should be available for the entire population of Berlin, two additional STEMOs have been implemented and gone operational in 2017 and 2018 – under the condition of an accompanying scientific evaluation on outcome effects.

### Eligibility criteria

All patients calling the emergency services and prompting a stroke alarm [9] at the dispatch center will be screened for eligibility. Only treatment candidates defined by the inclusion and exclusion criteria listed in Table 1 will be included in the primary study population and thus compared for the primary outcome. Thereby, the screening process is operationalized aiming at a minimization of selection bias. Monitoring is carried out continuously for all eligibility criteria. If ascertainment is uncertain, the clinical documentation is submitted to an independent adjudication committee for blinded judgement. Additionally, patients with [1] stroke mimics receiving thrombolysis will be included in a sensitivity analysis together with the primary study population, and patients with [2] intracerebral hemorrhage presenting within 6 h from symptom onset will be analyzed as a companion study population.

**Table 1:** Inclusion and exclusion criteria for the study population

Inclusion criteria:	
1.	Suspected acute stroke according to the dispatcher stroke identification algorithm [8] during MSU hours and within the MSU catchment area
2.	Age ≥ 18 years
Inclusion criteria for the primary study population:	
3.	Final diagnosis of ischemic stroke (ICD-10: I63) or TIA (G45 except G45.4)
4.	Pre-stroke mRS ≤ 3 (able to ambulate without assistance)
5.	Confirmed onset-to-alarm time ≤ 4 h
Exclusion criteria:	
1.	Symptom remission until arrival of MSU or regular ambulance^a^
2.	Malignant or other severe primary disease with life expectancy < 1 year
Exclusion criteria for the primary study population:	
3.	Major surgery within last 4 weeks
4.	Confirmed stroke within last 3 months
5.	Absolute contraindication for both thrombolysis and endovascular treatment

*ICD-10* International Statistical Classification of Diseases and Related Health Problems 10th revision, *mRS* modified Rankin Scale, *MSU* Mobile Stroke Unit, *TIA* transient ischemic attack, ^a^no acute disabling neurological symptoms described in emergency medical service (EMS) documentation

### Outcome measures

The primary outcome is the modified Rankin score (mRS) 3 months after the acute event which is the most common outcome measure in stroke trials [12]. Since the type of intervention does not allow blinding of patients, the structured telephone interviews after 3 months are recorded and subsequently assessed by stroke experts who are unaware of the treatment arm allocation. For those patients who remain unreachable via phone or mail, we use information from registration offices regarding vital and residential status including living address. This information allows assessment of the co-primary outcome consisting of the following three categories: ‘1’ able to ambulate (mRS 0–3) or (if mRS not available) living at home, ‘2’ living with severe disability (mRS 4–5) or living in institutional care, and ‘3’ death (mRS 6). We introduced this novel outcome definition within a study protocol amendment during the first part of the B_PROUD trial in order to use all available information for the purpose of informing parties involved in acute stroke care organization on the effectiveness of the MSU concept. Only if the lost-to-follow-up rate is higher than 9% in this community nested trial, this outcome will be used as a co-primary outcome measure. .

Secondary outcomes include performance measures like thrombolysis and endovascular treatment rates and process times as well as clinical outcomes such as quality of life and dichotomized three-months mRS. All outcome measures are listed in in detail in Table 2.

**Table 2:** Outcome measures

Primary outcome measure:	
1.	Primary outcome: assessment of functional outcome over the entire range of the mRS
2.	Co-primary outcome: assessment of functional outcome including the following range of outcomes: mRS 0–3 if available, mRS 4–5 or (if mRS is missing) living in institution (information according registration office at 4 months after stroke), and death
Secondary outcome measures:	
1.	Thrombolysis rate
2.	Endovascular thrombectomy rate
3.	Onset-to-treatment time
4.	Onset-to-reperfusion time (for endovascular thrombectomy, Charité centers only)
5.	Alarm-to-imaging time
6.	Alarm-to-treatment time
7.	Imaging-to-treatment time
8.	Cost effectiveness (additional costs due to implementation and running of STEMO, duration of hospital stay regarding acute treatment and rehabilitation, hospital related costs, costs of long-term care, and combination of above mentioned)
9.	Quality of life (EQ-5D)
10.	Shift analyses for mRS ≤ 1 at 3 months in patients ≤80 years of age living at home without disability and mRS ≤ 2 at 3 months in patients > 80 years of age living at home with help or living in an institution
11.	Secondary ICH after thrombolysis or thrombectomy
12.	Symptomatic secondary ICH according to the discharge letter
13.	In-hospital mortality
14.	Death rate over time (Kaplan-Meier plot)
15.	Discharge status (including in-hospital mortality among patients not included in the primary study population, especially patients with ICH)
16.	Functional outcome among patients with ICH
17.	Rate of emergency medical service deliveries to specialized facilities (patients with large vessel occlusion to endovascular thrombectomy capable facility, patients with ICH to neurosurgery department)

*EQ-5D* EuroQol Group 5 dimensions, *ICD-10* International Statistical Classification of Diseases and Related Health Problems 10th revision, *ICH* intracerebral hemorrhage, *mRS* modified Rankin Scale, *MSU* Mobile Stroke Unit, *TIA* transient ischemic attack

A continuous reporting system for serious adverse events of special interests (SAESI) is implemented to check rates of symptomatic secondary intracerebral hemorrhages (sICH) or deaths. In case of more than 10 deaths within 7 days (or at discharge, whatever comes first), or more than 10 symptomatic sICH per 100 treatment candidates in the MSU group, the study has to be stopped after recommendation by an external data safety monitoring board. To address the intention-to-treat approach for outcome analyses, we rigorously consider STEMO availability as determinative and, for example, count cancellations of STEMO dispatches to the MSU intervention group.

### Sample size estimation

The proposed sample size to be analyzed is 1372 patients – equal to the sample size of B_PROUD part 1. Sample size calculation was originally performed in October 2015 based on outcomes seen in a registry-based comparison of the first 193 patients with pre-hospital thrombolysis on STEMO and 615 consecutive patients with in-hospital thrombolysis of the Charité thrombolysis registry. Inclusion of 1500 patients will be necessary, considering 9% lost-to-follow-up. For the primary outcome, we expect the following differences (STEMO vs. control group): mRS 0: 21/21%; 1: 21/15%; 2: 7/9%; 3: 20/12%; 4: 11/14%; 5: 5/9%; 6: 15/20%. The Mann-Whitney test with two-sided significance level of 0.05 has 80% power to detect such a group difference in at least 686 patients per group.

In case of more than 9% lost-to-follow-up, the study will be seen as successful only if both co-primary endpoints (mRS, mRS in three categories: 0–3, 4–5, 6) show significant better outcome for the intervention using a two-sided significance level of *α* = 0.05.

The power calculation was conducted with the R-Package sample size [13]. Since there is scarce information on the possible effect size, an interim analysis for a blinded sample size re-estimation is planned after primary outcome assessment of 300 patients.

### Contacts

B_PROUD is sponsored by Charité – University Medicine Berlin, Berlin, Germany via the Center for Stroke Research Berlin and the excellence cluster NeuroCure, Berlin, Germany, and funded by German Research Foundation. The B_PROUD MSU evaluation is conducted in close collaboration with the Berlin Fire Department and its medical lead of EMS as well as with the 15 Berlin stroke centers.

## Perspective

While scientific evaluations of the Berlin MSU implementation so far have shown that pre-hospital thrombolysis is safe and associated with a substantial shortening in time to treatment [4, 7], a definite proof of clinical benefit has not yet been accomplished. The B_PROUD trial was therefore designed to provide confirmatory evidence that earlier (pre-hospital) stroke work-up and treatment leads to better functional outcomes. Recruitment to B_PROUD part 1 was started in February 2017 and is planned to be completed in 2019, concurrent with a similar trial, the BEST-MSU trial [14]. Despite similar inclusion criteria of the latter also ensuring the comparison of only treatment candidates, our study design allows follow-up of the patients without written informed consent which may to some extend limit external validity by introducing a selection bias.

In addition to the primary outcome, the B_PROUD study will provide valuable information on effects of earlier treatment (blood pressure lowering and anticoagulation reversal) on hematoma volumes in intracerebral hemorrhages.

While the time saving approach of the prehospital MSU concept is intriguing, stakeholders of stroke care need reliable information on the effect size of clinical outcomes and cost-effectiveness. The expected benefits in outcome and potentially reduced costs for hospital and long-term care need to be weighed against additional costs of implementation and running not only a single but three STEMOs. B_PROUD and parallel studies such as BEST-MSU [14] are designed to provide the needed data and will therefore support future decision making.

## Abbreviations

B_PROUD: Berlin Prehospital Or Usual Delivery of acute stroke care
BEST-MSU: Benefits of Stroke Treatment Delivered Using a Mobile Stroke Unit
B-SPATIAL: Berlin - specific acute therapy in ischemic or hemorrhagic stroke with long-term follow-up
CT: Computed tomography
EMS: Emergency medical service
EQ-5D: EuroQol Group 5 dimensions
ICD10: International Statistical Classification of Diseases and Related Health Problems 10th revision
ICH: Intracerebral hemorrhage
mRS: Modified Rankin scale
MSU: Mobile Stroke Unit
PHANTOM-S: Pre-Hospital Acute Neurological Therapy and Optimization of Medical care in Stroke patients
PRESTO: Pre-hospital Stroke Treatment Organization
PROBE: Prospective randomized open blinded end-point
SAESI: Serious adverse events of special interests
sICH: secondary intracerebral hemorrhage
STEMO: Stroke Emergency Mobile
TIA: Transient ischemic attack
